# Pyruvate Kinase M2 and Lactate Dehydrogenase A Are Overexpressed in Pancreatic Cancer and Correlate with Poor Outcome

**DOI:** 10.1371/journal.pone.0151635

**Published:** 2016-03-18

**Authors:** Goran Hamid Mohammad, S. W. M. Olde Damink, Massimo Malago, Dipok Kumar Dhar, Stephen P. Pereira

**Affiliations:** 1 UCL Institute for Liver and Digestive Health, Royal Free Hospital Campus, University College London, London, United Kingdom; 2 Chemistry Department, School of Science, University of Sulaimani, Sulaimanyah, Kurdistan Region, Iraq; 3 Department of Surgery, Maastricht University Medical Centre, Maastricht, the Netherlands; 4 King Faisal Specialist Hospital and Research Centre, Riyadh, Saudi Arabia; University Hospital Carl Gustav Carus Dresden, GERMANY

## Abstract

Pancreatic cancer has a 5-year survival rate of less than 4%. Despite advances in diagnostic technology, pancreatic cancer continues to be diagnosed at a late and incurable stage. Accurate biomarkers for early diagnosis and to predict treatment response are urgently needed. Since alteration of glucose metabolism is one of the hallmarks of cancer cells, we proposed that pyruvate kinase type M2 (M2PK) and lactate dehydrogenase A (LDHA) enzymes could represent novel diagnostic markers and potential therapeutic targets in pancreatic cancer. In 266 tissue sections from normal pancreas, pancreatic cystic neoplasms, pancreatic intraepithelial neoplasia (PanIN) and cancer, we evaluated the expression of PKM2, LDHA, Ki-67 and CD8+ by immunohistochemistry and correlated these markers with clinicopathological characteristics and patient survival. PKM2 and LDHA expression was also assessed by Western blot in 10 human pancreatic cancer cell lines. PKM2 expression increased progressively from cyst through PanIN to cancer, whereas LDHA was overexpressed throughout the carcinogenic process. All but one cell line showed high expression of both proteins. Patients with strong PKM2 and LDHA expression had significantly worse survival than those with weak PKM2 and/or LDHA expression (7.0 months vs. 27.9 months, respectively, p = 0.003, log rank test). The expression of both PKM2 and LDHA correlated directly with Ki-67 expression, and inversely with intratumoral CD8+ cell count. PKM2 was significantly overexpressed in poorly differentiated tumours and both PKM2 and LDHA were overexpressed in larger tumours. Multivariable analysis showed that combined expression of PKM2 and LDHA was an independent poor prognostic marker for survival. In conclusion, our results demonstrate a high expression pattern of two major glycolytic enzymes during pancreatic carcinogenesis, with increased expression in aggressive tumours and a significant adverse effect on survival.

## Introduction

Pancreatic cancer is one of the most common gastrointestinal cancers and the fourth most common cause of cancer-related deaths worldwide [1]. Surgical resection is the most effective therapy but patients are usually diagnosed at an advanced stage when surgical resection is not feasible, resulting in a five year survival rate of less than 4% [2]. There are few other effective therapies, with palliative single-agent or combination chemotherapy as the main treatment option for patients with advanced disease [3,4].

Aerobic glycolysis is a hallmark of cancer cells, with the production of lactate even in the presence of ample amounts of oxygen, a phenomenon known as the Warburg effect [5–7]. An important advantage of increased glycolysis in tumour cells is production of energy without the consumption of oxygen and glycolytic intermediates, such as amino acids, nucleotides, phospholipids and triglycerides, which are used as macromolecules for the synthesis of structural elements of new cells [5,6,8,9].

Pyruvate kinase (PK) is a tightly regulated glycolytic enzyme that catalyses the last step of glycolysis and mediates the transfer of phosphate from phosphoenolpyruvate (PEP) to adenosine diphosphate (ADP) to produce pyruvate and energy (ATP) [10–12]. PK has four different isoenzymes PKM1, PKM2, PKL and PKR, the expression of which depends on the metabolic response of cells. Both L and M genes encode PK isoenzymes. The L gene encodes both L and R-PK isoenzymes, while the M gene encodes both M1 and M2 isoenzymes (PKM2) [13–17]. During tumorigenesis, the expression of specific PK isoenzymes, for instance PK-M1 in the brain and PK-L in the liver, disappears and PKM2 expression predominates [18]. PKM2 expression can oscillate between the highly active tetrameric isoform and the nearly inactive dimeric isoform, depending on the cellular demand of energy or production of anabolic intermediates for cell proliferation. The tetrameric isoform of PKM2 is predominantly expressed in normal cells, while its dimeric isoform is usually found in tumour cells, hence the name tumour PKM2 [11,14,19].

Another downstream component in the glycolytic pathway is the lactate dehydrogenase A (LDHA) enzyme, which is a part of the LDH family of 2-hydroxyacid oxidoreductases. LDHs are homo- and hetero- tetrameric enzymes comprised of two major subunits, A and B, resulting in five isoenzymes that catalyse the reversible conversion of pyruvate and lactate. LDHA (also known as LDH-5, LDH-M or A4) is encoded by the *ldh-a* gene and predominates in skeletal muscle and liver, while LDHB (also known as LDH-1, LDH-H or B4) is encoded by the *ldh-b* gene and is found mainly in the heart and brain [20–24]. LDH-C is mainly composed of X-subunits and is found in human spermatozoa [22,25]. LDHA catalyses the conversion of pyruvate into lactate with regeneration of NAD^+^ to continue energy production by glycolysis [26–29]. Lactate produced by LDHA is used as an alternative fuel by cells that are adjacent to blood vessels, and glucose is spared for more distant cells that are hypoxic.

Overexpression of PKM2 or LDHA has been reported in the tissues of a number of cancers, including cholangiocarcinoma, colorectal cancer, non-small cell lung cancer and pancreatic cancer. Overexpression is associated with tumour initiation, progression and resistance to chemotherapy [27,30–32]. In theory, the expression profile of these enzymes may also represent useful diagnostic or prognostic markers in pancreatic cancer [15,23,33].

CD8+ T cells (Cytotoxic T lymphocytes) are an important subset of tumour infiltrating lymphocytes that play a key role in the anti-tumour immune response. Immunohistochemistry studies have shown an anti-tumour effect of infiltrating CD8+ lymphocytes, with improved survival rates of patients with pancreatic, lung, ovarian, colorectal, renal and oesophageal tumours [34–39], and a direct correlation between increasing number of CD8+ tumour infiltrating lymphocytes (TILs) and tumour cell apoptosis [40,41].

The aim of our study was to evaluate the expression of PKM2 and LDHA in pancreatic pre-neoplastic lesions (cysts and pancreatic intraepithelial neoplasia, PanIN) and cancers and to correlate it with patient outcome. Given that PKM2 and LDHA are involved in several cell proliferation signaling pathways [42], we also investigated whether the expression of these glycolytic enzymes correlated with the number of CD8+TILs and markers of tumour proliferation (Ki-67).

## Materials and Methods

### Patients

This study included pancreatic biopsy or surgical resection specimens from 266 patients; 136 from University College London Hospital NHS Foundation Trust (UCLH) and another 130 from a commercially available tissue microarray (TMAs) (AccuMaxArray, ISU ABXIS CO., LTD, USA, and Insight Biotechnology Limited, UK). The UCLH cohort consisted of 136 patients with confirmed pancreatic ductal adenocarcinoma (PDAC) (n = 61), ampullary adenocarcinoma (n = 11), pancreatic cystic lesions (n = 49), chronic pancreatitis (n = 11) and normal pancreatic tissue (n = 4). These patients had been treated at UCLH between January 2005 and January 2010. A clinical database of the UCLH patients was created through the CoPath histology database, which included the following clinicopathological parameters: gender, age at diagnosis, sample type (biopsy or resection), type of tumour (ampullary or ductal), outcome (alive or dead), cause of death, length of follow-up if alive, presence or absence of metastatic disease at the time of histological diagnosis, whether resection was carried out, residual disease (R) status, post-resection recurrence, time to post-resection recurrence (if any), cancer stage, lymph node involvement, presence of perineural or lymphovascular invasion, degree of tumour differentiation, and whether patients received chemotherapy (Table 1). All data were collected from the date of histological diagnosis to the date of death or the end of data collection on 1 February 2015. Ethics committee approval was obtained from the Central London REC 3 Research Ethics Committee to perform immunohistochemistry on stored biopsy and resection specimens linked with a clinical database with the need for consent waived (REC reference 06/Q0512/106, amendment date 30 July 2010). All patient samples were anonymised and de-identified prior to analysis.

**Table 1:** Correlation of PKM2 and LDHA expression with clinicopathological factors (UCLH cohort).

Variables	Total (n = 72)	PKM2 Expression		*P* value	LDH-A Expression		*P* value	Combined expression		*P* value
		Positive	Negative		Positive	Negative		Positive*	Negative**	
		(High)	(Low)		(High)	(Low)		(High)	(Low)	
		(*n* = 46)	(*n* = 26)		(*n* = 55)	(*n* = 17)		(*n* = 44)	(*n* = 28)	
		*n* (%)	*n* (%)		*n* (%)	*n* (%)		*n* (%)	*n* (%)	
**Tumour Types**										
PDAC	61	41 (89)	20 (77)	0.171	48 (87)	13 (76.5)	0.249	40 (91)	21 (75)	0.088
Ampullary	11	5 (11)	6 (23)		7 (13)	4 (23.5)		4 (9)	7 (25)	
**Sex**										
Male	39	25 (54.4)	14 (53.8)	0.968	29 (52.7)	10 (58.8)	0.46	21 (47.7)	18 (64.3)	0.116
Female	33	21 (45.6)	12 (46.2)		26 (47.3)	7 (41.2)		23 (52.3)	10 (35.7)	
**Age at Diagnosis (years)**										
Mean ± SD	72	63.2±11.9	65±9.4	0.502	63.8±11.1	64±11.6	0.959	62.7±11.4	65.6±10.4	0.339
**Tumour Differentiation**										
Well/Mod	18	8 (32.5)	10 (77.5)	0.047	13 (26)	5 (74)	0.63	8 (18.2)	10 (35.7)	0.094
Mod/Poor	54	38 (67.5)	16 (32.5)		42 (74)	12 (26)		36 (81.8)	18 (64.3)	
**Metastasis Status**										
Patients with metastasis	20	15 (32.6)	5 (19.3)	0.229	15 (27.3)	5 (29.4)	0.942	15 (34.1)	5 (17.8)	0.104
Patients without metastasis	52	31 (67.4)	21 (80.7)		40 (72.7)	12 (70.6)		29 (65.9)	23 (82.2)	
**Lymph Node Involvement**										
Positive lymph node	16	10 (21.7)	6 (23)	0.504	13 (23.6)	3 (17.6)	0.942	9 (20.5)	7 (25)	0.444
Negative lymph node	11	6 (13)	5 (19.3)		8 (14.5)	3 (17.6)		5 (11.4)	6 (21.4)	
Unknown	45	30 (65.3)	15 (57.7)		34 (61.9)	11 (64.7)		30 (68.1)	15 (53.6)	
**Clinical T-Stage Classification**										
T1	0	0 (0)	0 (0)	0.615	0 (0)	0 (0)	0.402	0 (0)	0 (0)	0.358
T2	7	3 (6.5)	4 (15.4)		4 (7.3)	3 (17.6)		3 (6.9)	4 (14.3)	
T3	11	7 (15.2)	4 (15.4)		10 (18.2)	1 (5.9)		7 (15.9)	4 (14.3)	
T4	9	6 (13)	3 (10.7)		7 (12.7)	2 (11.8)		4 (9.1)	5 (17.8)	
Unknown	45	30 (62.3)	15 (53.5)		34 (61.8)	11 (64.7)		30 (68.1)	15 (53.6)	
**Staging**										
Stage I	3	1 (2.2)	2 (7.7)	0.503	1 (1.8)	2 (11.8)	0.2	2 (4.6)	1 (3.5)	0.264
Stage II	13	7 (15.2)	6 (23)		11 (20)	2 (11.8)		6 (13.6)	7 (25)	
Stage III	9	6 (13)	3 (11.5)		7 (12.7)	2 (11.8)		4 (9.1)	5 (17.9)	
Stage IV	2	2 (4.4)	0 (0)		2 (3.6)	0 (0)		2 (4.6)	0 (0.0)	
Unknown	45	30 (65.2)	15 (57.8)		34 (61.9)	11 (64.6)		30 (68.1)	15 (53.6)	
**Mean of CD8/HPF**										
Mean ± SD	72	16.3±24.3	42.4±43.1	**0.0001**	20.1±30.7	44.3±40.1	**0.005**	16.4±24.9	39.5±41.7	**0.001**
**Ki67 Proliferation Index (%)**										
Mean ± SD	72	27.8±12.9	12.2±14	**0.0001**	25.1±14	12.3±15.1	**0.004**	27.8±12.8	13.7±14.7	**0.0001**

* Both PKM2 and LDH-A positive

** Either PKM2 or LDH-A and both negative

### Tissue microarray

A second validation set of 130 tissue samples was derived from commercially available pancreatic TMAs. A total of 206 tissue cores from 130 patients (83 male, 47 female, median age 59, range 32–80 years) were present on the TMAs, which included 63 cases of PDAC in duplicate, 19 PanIN (10 PanIN-1, 7 PanIN-2 and 2 PanIN-3), 10 chronic pancreatitis and 38 normal pancreatic tissue cores. Histological diagnosis of all cases was verified by haematoxylin and eosin staining. Clinical data provided with the TMAs included tumour size and stage, degree of tumour differentiation, lymph node involvement, metastatic status and tumour sites.

### Immunohistochemistry

Standard immunohistochemistry methods were used to detect the expression of PKM2, LDHA, CD8 and Ki-67. Briefly, paraffin embedded sections were pre-heated in an oven for 30 minutes at 60°C, and then deparaffinised in xylene and hydrated through a series of graded ethanol (70%-95%). The sections were subjected to heat-mediated antigen retrieval with citrate buffer (pH 4.0) in an autoclave. Endogenous peroxidase activity was inhibited by 3% hydrogen peroxide for 20 minutes, followed by incubation with 2.5% normal horse blocking serum for 20 minutes. The sections were then incubated with primary antibody for one hour at room temperature for CD8 (CD8A Rabbit polyclonal antibody, Abnova, UK, Cat# PAB11235, 1:200) and Ki-67 (Rabbit polyclonal to Ki-67, Abcam, UK, Cat# ab15580, 1:300), and overnight at 4°C for PKM2 (Monoclonal mouse anti-human and rat PKM2, ScheBo®Biotech, Giessen, Germany, Cat# S-1, 1:100) and LDHA (LDHA/LDHC (C28H7) Rabbit Monoclonal antibody, Cell Signaling, UK, Cat #3558, 1:250) in PBS. After three washes with PBS containing 0.5% Tween 20, the sections were incubated with horseradish peroxidase (HRP)–conjugated secondary antibody for 30 minutes. Primary antibody was detected using the 3, 3’-diaminobenzidine (DAB) or 3-amino-9-ethyl-carbazole (AEC) detection system kit. The sections were placed in haematoxylin for 3 minutes, then gently washed and mounted.

### Double immunohistochemistry

A double immunostaining kit (PicTure double staining kit, Invitrogen, UK) was used for co-localisation of PKM2 and CD8 or Ki-67. Briefly, two different enzyme detection systems were used for the sequential double staining with initial use of the horseradish peroxidase system followed by the alkaline phosphate system. Sections were incubated with the anti-PKM2 primary antibody and HRP secondary antibody, and visualisation was developed with the DAB detection system. Following this step, sections were washed with PBS, incubated with blocking serum, followed by primary antibody (anti-CD8 or Ki-67) and Goat anti-Rabbit IgG alkaline phosphatase secondary antibody. Colour was developed by fast red with counter staining by haematoxylin.

### Evaluation of immunohistochemistry staining

Immunohistochemistry was evaluated using a conference light microscope (AXIO Scope, Carl Zeiss MicroImaging GmbH, Gottingen, Germany). The assessment of the immunostained slides was performed independently by two observers, blinded to patient background information, and any disparity between the observers was resolved by using a conference microscope. A comprehensive scoring formula was used for the semi-quantitative evaluation of PKM2 expression as described previously [43], with intensity of staining scored as: 1, weak expression; 2, moderate expression; or 3, strong expression; the extent of staining scored as 1, <33% of tumour cells positive; 2, 33–67% of tumour cells positive or; 3, >67% of tumour cells positive. The intensity and extent scores were then multiplied to obtain a single scale of scores of 1, 2, 3, 4, 6, and 9. The scores of 1–3 were defined as weak (or negative) staining, whereas scores 4, 6 and 9 were considered as strong (or positive) staining.

The numbers of CD8+ TILs and Ki-67 positive tumour cells were also counted independently by two observers. Initially, the whole slide was scanned at low magnification (x40) to identify the region with the highest density of intratumoural CD8 or Ki-67-positive tumour cells and then five random areas within that region were counted at high magnification (x400). The average number of CD8+ TILs was calculated and expressed as count per high power field (HPF). The cell proliferation index (PI) was expressed as a percentage of the number of nuclei stained positive for Ki-67 among 1000 tumour cells using a standardised grid.

### Cell cultures

Nine human pancreatic cancer cell lines were purchased from RIKEN BioResource Centre (RIKEN BRC, Tsukuba, Japan) and the BxPc-3 cell line was purchased from PerknElmer (Caliper LifeSciences, Hopkinton, MA, USA). PANC-1, PK-1, PK-59, PK-45H, PK45P, KLM-1, NOR-P1 and BxPc-3 were maintained in RPMI-1640, Miapaca-2 in DMED and KP-4 in DMEM/F12 medium. All media were supplemented with 10% fetal bovine serum (FBS), 1% penicillin/streptomycin and 2mM glutamine (Gibco, Life technologies, UK). Cells were maintained in a humidified atmosphere of 21% O_2_, 5% CO_2_ at 37°C and harvested with trypsin-EDTA.

### Western Blot

PKM2 and LDHA expression was evaluated in pancreatic cancer cell lines by Western Blotting. Briefly, following protein extraction, protein concentration was measured by the Bicinchoninic Acid (BCA) assay and 15μg protein was run on a pre-cast gel (NuPAGE Novex 4–12% Bis-Tris 1.0mm, 10 wells gel, Invitrogen, USA) and transferred onto a 0.45μm pore size Polyvinylidene fluoride (PVDF) membrane (Invitrogen, USA). The membrane was blocked with 5% Bovine Serum Albumin (BSA) solution and incubated overnight at 4°C either with mouse anti-PKM2 (DF-4, ScheBo®Biotech, Giessen, Germany, 1:1000) or rabbit anti-LDHA antibody (Cell Signaling, UK, 1:1000), and then incubated with appropriate horseradish peroxidase conjugated secondary antibodies (Cell Signaling, UK, 1:2000). The antigen antibody reaction was detected by enhanced chemiluminescence substrate (Thermo Scientific, USA). Anti-β actin antibody (Cell Signaling, UK) was used as a protein loading control.

### Statistical analysis

IBM SPSS Statistical software (Version 22, SPSS Inc., Chicago, IL, USA) was used for data analysis and graphics. Kaplan-Meier plots and log-rank tests were used to analyse survival and to identify differences between groups. One way ANOVA with Bonferroni post-hoc test was used for overall comparison of multiple groups, with the Mann-Whitney U-test for nonparametric tests and the Chi square test for differences between categorical data. Non-parametric correlation analyses between two continuous variables were performed by Spearman test. All test results were two-tailed, with effects summarised using 95% confidence intervals. Statistical significance was set at p<0.05.

## Results

### Expression of PKM2 and LDHA in pancreatic cancer

**Fig 1:**
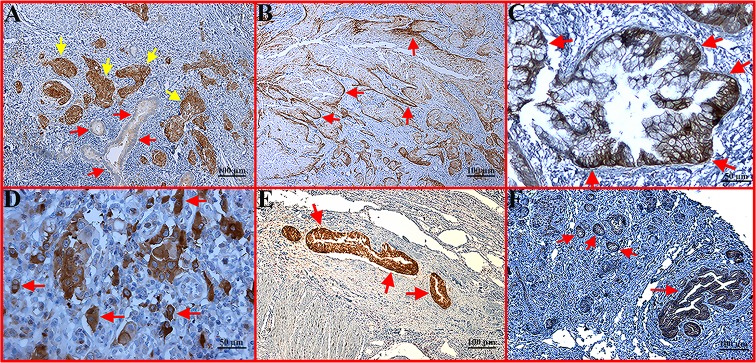
Immunohistochemical staining of PKM2 expression in representative pancreatic tumour sections. (A) Well differentiated area of tumour showing weak PKM2 expression (red arrow), with high expression in poorly differentiated areas (brown colour, yellow arrow) (x100 magnification). (B) Growing margin of tumour nodules with strong expression of PKM2 (red arrow) (x100 magnification). (C) Membranous expression of PKM2 (red arrow) (x200 magnification). (D) Heterogeneous expression of PKM2 with predominant expression in the proliferating cells (red arrow) (x200 magnification). (E) Strongly positive tumour expression of PKM2 in vascular invasion (red arrow) (x100 magnification). (F) Strongly positive tumour expression of PKM2 with muscular invasion (red arrow) (x100 magnification).

**Fig 2:**
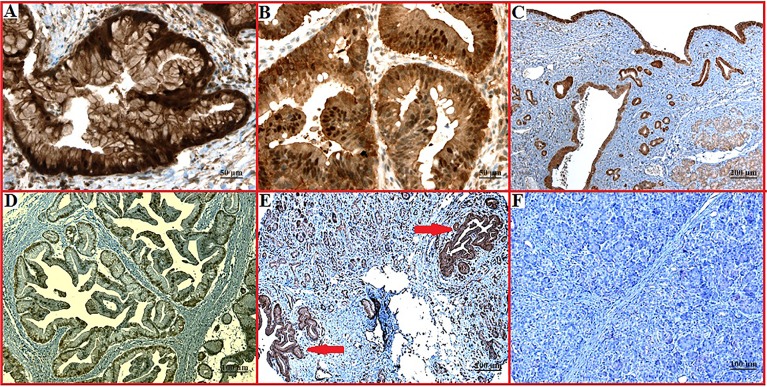
LDHA expression pattern in pancreatic cancer, benign and normal tissue sections. (A) Membranous expression of LDHA (x200 magnification). (B) Cytoplasmic and nuclear expression (x200 magnification). (C) Strong expression in pancreatic cyst with mild expression in the surrounding normal pancreatic tissue (x50 magnification). (D) Nuclear expression of LDHA in pancreatic cancer (x100 magnification). (E) Strong expression in PanIN lesion (x100 magnification). (F) Negative staining in normal pancreas (x100 magnification).

Both PKM2 and LDHA were overexpressed in tumour cells compared with normal pancreatic tissue. A variable expression pattern of PKM2 was observed in tumour tissues, with relatively higher expression in poorly differentiated areas, in advancing margins of tumour nodules and in invasive (muscular and blood vessel) tumours (Fig 1A, 1B, 1E and 1F). Overall, PKM2 expression was predominantly associated with aggressive tumours. Preferential expression of PKM2 was observed in binucleated proliferating cells in tumour nodules (Fig 1D). Expression of PKM2 was noted in all cell compartments, including the cell membrane, cytoplasm and/or nucleus (Fig 1C and 1F). In contrast, LDHA expression was generally high in tumour as well as in preneoplastic tissues and pancreatitis without a specific pattern (Fig 2). LDHA expression was also detected in the cell membrane and/or cytoplasm and occasionally in the nucleus.

**Fig 3:**
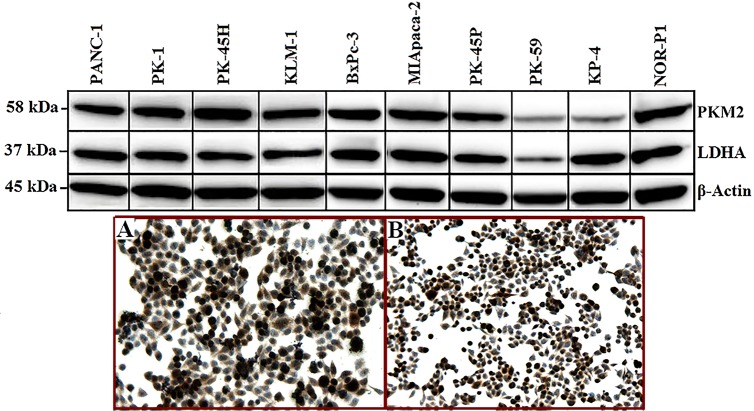
Expression of PKM2 and LDHA in pancreatic cancer cell lines as detected by Western blot analysis (upper panel). Immunostaining of Miapaca-2 cells with PKM2 (A) and LDHA (B) is shown in the lower panel. Strong cytoplasmic and nuclear staining is noted in proliferating cells.

**Fig 4:**
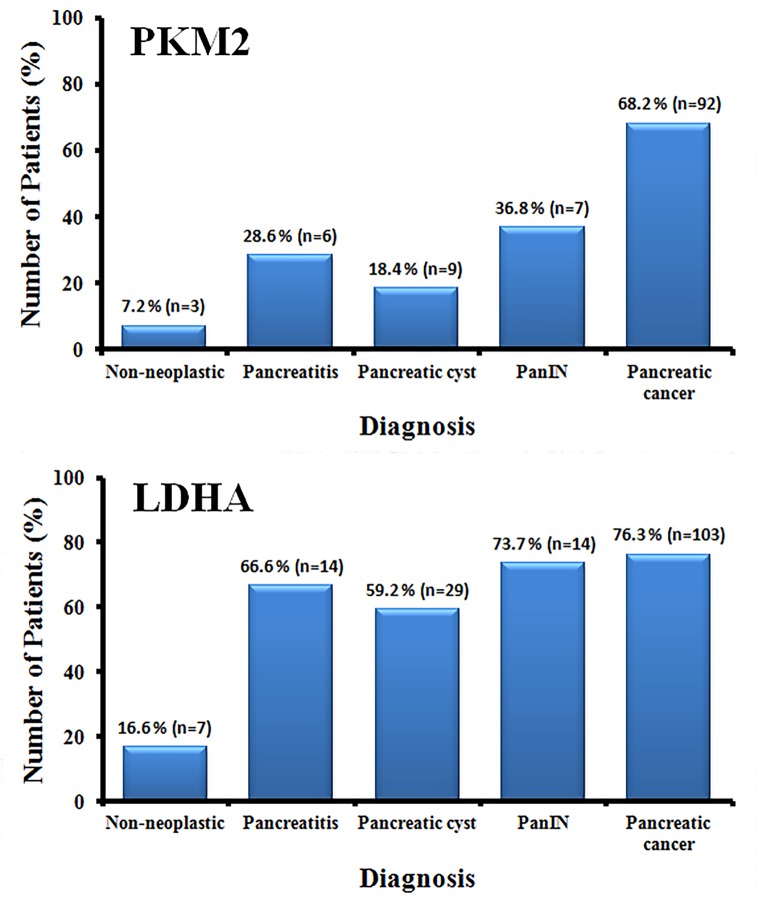
Percentages of PKM2 and LDHA expression in different tissue types. PKM2 was strongly expressed by pancreatic cancer tissue specimens and was significantly higher than in normal, pancreatitis, pancreatic cyst and PanIN tissues (P<0.001). Expression of LDHA was significantly higher in pancreatic cancer than in normal pancreas (P<0.001), whereas, there was no significant differences in LDHA expression between pancreatic cancer, PanIN, pancreatic cysts and pancreatitis.

Similar expression of PKM2 and LDHA was observed in the UCLH cohort and TMA samples, with a staining score of > 3 in 64% and 73% of tumours, respectively, for PKM2, and in 76% of tumours for LDHA in both cohorts. The expression pattern in the pancreatic cancer cell lines was similar for both PKM2 and LDHA, except in the KP4 cell line in which the LDHA level was stronger than PKM2 level (Fig 3). In both cohorts, pancreatitis samples also highly expressed LDHA compared with PKM2 expression (Fig 4).

As shown in Fig 4, progressively higher PKM2 expression was observed along the transition to pancreatic cancer, with the lowest expression in pancreatic cysts (19%), intermediate in PanIN (37%) and highest in cancers (68%). PKM2 expression was approximately four-fold higher in pancreatitis (29%) compared with normal pancreatic tissue. Although LDHA expression was also significantly increased in cancers compared with normal pancreatic tissue (p < 0.0001, ANOVA), there were no significant differences between chronic pancreatitis, pancreatic cysts, PanIN and cancer samples (67%, 59%, 73% and 76%, respectively).

### PKM2 and LDHA expression in pancreatic cancer cell lines

By Western blotting, high expression of PKM2 and LDHA was detected in 8 and 9 out of the 10 pancreatic cancer cell lines (Fig 3). The expression level of PKM2 and LDHA corresponded in all cell lines except in the KP4 in which the PKM2 level was weaker than the LDHA level. By immunohistochemistry, both PKM2 and LDHA had strong cytoplasmic and nuclear expression (Fig 3).

### Association with clinicopathological parameters

The correlation between PKM2 and LDHA expression with clinicopathological characteristics is shown in Table 1. There was a significant inverse correlation between PKM2 expression and tumour differentiation in the UCLH cohort, with 83% of PKM2 positive tumours being less differentiated compared with 64% of PKM2 negative tumours (p = 0.047, Chi-square test) (data not shown).

**Fig 5:**
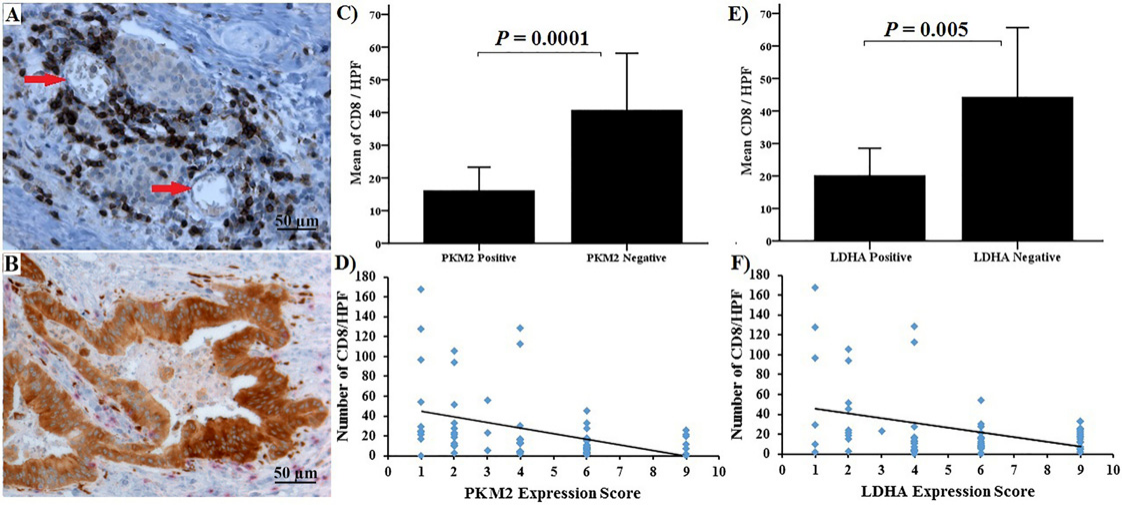
Immunohistochemical staining and correlation between PKM2 and LDHA and CD8+TIL. (A) Well differentiated tumours with negative PKM2 expression had strong infiltration by CD8+ positive T-lymphocytes (x200 magnification). (B) Poorly differentiated tumours strongly positive for PKM2 had sparse infiltration by CD8+ positive T-lymphocytes (x200 magnification). There was a significant negative correlation between CD8+ positive cells and both PKM2 (C) & (D) and LDHA staining (E) & (F).

**Fig 6:**
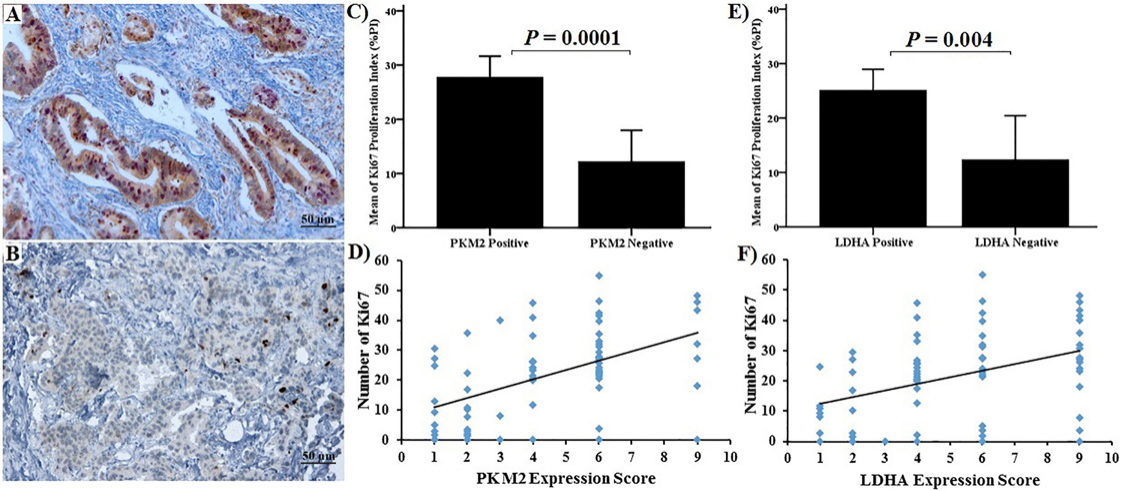
Immunohistochemical staining and correlation between PKM2, LDHA and Ki-67. (A) Tumours with strongly positive PKM2 expression had most of the nucleus stained for Ki-67 (x100 magnification). (B) Tumours weakly positive for PKM2 had scant Ki67 staining (x100 magnification). (C) & (D) Correlation between PKM2 staining and Ki-67 positive cells. (E) & (F) Correlation between LDHA staining and Ki-67 positive cells. There was a significant correlation for both PKM2 and LDHA.

A significantly higher number of CD8+ TIL was found in tumours with weak PKM2 or LDHA expression compared with tumours that had a strong expression (p = 0.0001, p = 0.005 respectively, Table 1). Furthermore, a significant association between tumour cell proliferation and expression of both PKM2 and LDHA was observed; the number of tumour nuclei expressing Ki-67 was more than 2-fold higher in PKM2 and LDHA expressing tumours compared with negative tumours (PKM2: 27.8 ± 12.9 vs. 12.2 ± 14, p = 0.0001 and LDHA: 25.1 ± 14 vs. 12.3 ± 15.1, p = 0.004). When staining scores, CD8+ TIL count and the number of Ki-67 positive cells were considered as continuous variables, a significant inverse correlation between the staining scores and CD8+ cell count was observed (PKM2: p<0.001 and LDHA: p = 0.004, Spearman rank correlation). A significant direct correlation was noticed between the staining scores and Ki-67 count (PKM2: p<0.001 and LDHA: p = 0.001, Spearman rank correlation test) (Figs 5 and 6). In the TMA cohort, the expression of PKM2 and LDHA correlated with tumour size. PKM2 expression was observed in 54.5%, 77.8% and 90.9% of tumours that were ≤ 2.5, 2.6–3.9 and ≥ 4 cm in size, respectively. Positive LDHA expression was found in 59.1%, 77.8% and 100% in tumours that were ≤ 2.5, 2.6–3.9 and ≥ 4 cm in size, respectively. There were no significant differences between PKM2 or LDHA expression and tumour location, lymph node involvement, T-stage and metastatic status.

### Correlation between PKM2 and LDHA expression and patient survival

**Fig 7:**
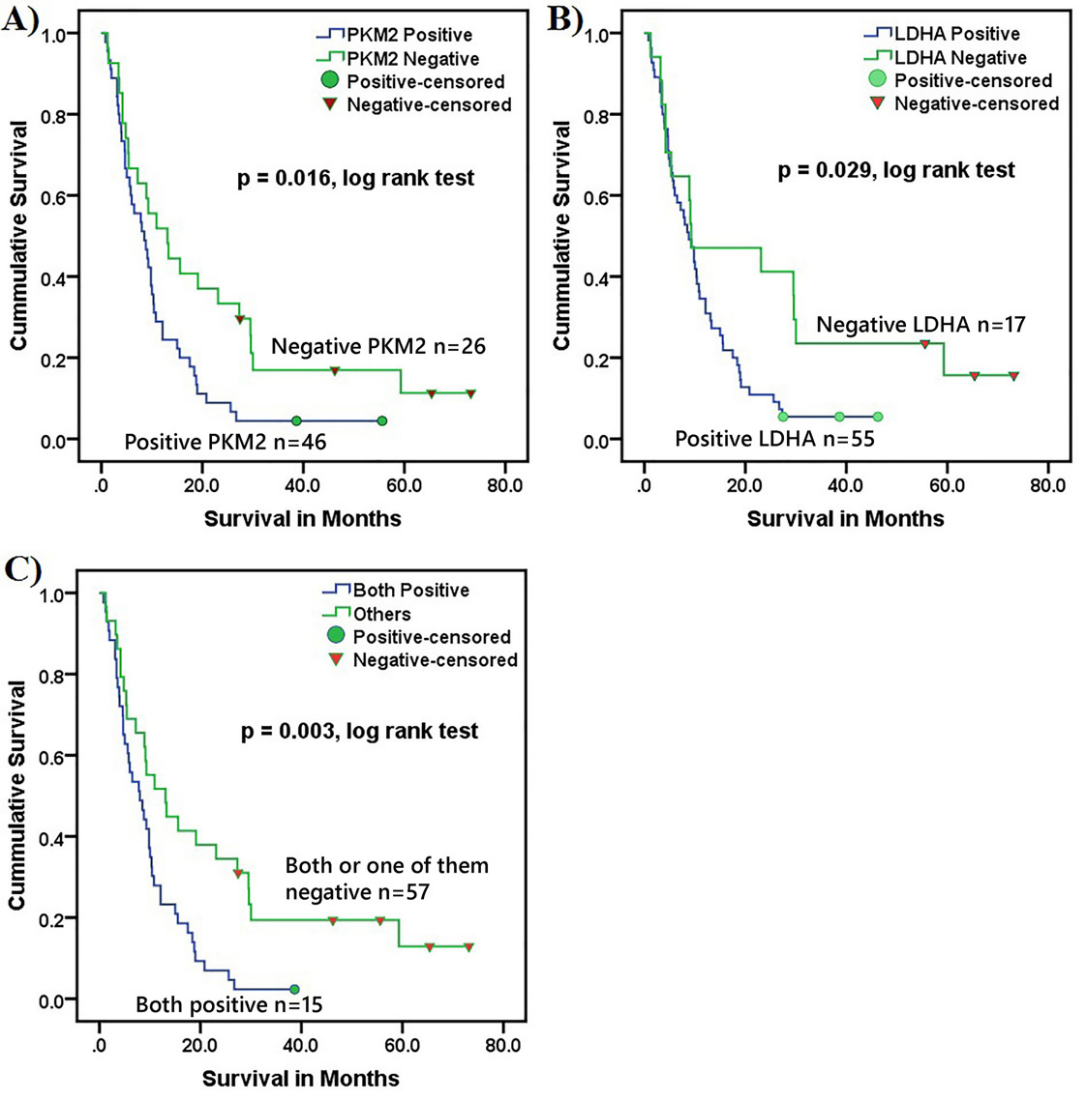
Overall patient survival in relation to PKM2 and LDHA expression. Both PKM2 (A) and LDHA (B) had a significant prognostic impact on patient survival (p = 0.016, 0.029, log rank test, respectively) and the combined expression for both markers further stratified the patients (C) (p = 0.003, log rank test). As expected, patients who underwent surgical resection had a longer survival than unresected patients.

We next examined whether expression profile of PKM2 and LDHA predicted survival. Patients with tumours scoring > 3 for PKM2 or LDHA expression had significantly worse survival compared with those that weakly expressed PKM2 and/or LDHA. Of the 72 pancreatic cancer samples (UCLH cohort), 46 (64%) strongly expressed PKM2 and these patients had a median survival of only 8.9 months compared with 28.9 months in the 26 (36%) patients with weak (negative) PKM2 tumour expression (p = 0.016, log-rank test, Fig 7). Similarly, 55 (76.4%) patients with positive LDHA tumour expression had a median survival of 10.9 months compared with 34.5 months of the 17 (23.6%) patients with weak (negative) LDHA tumour expression (p = 0.029, log-rank test, Fig 7). Moreover, when the expression profile of PKM2 and LDHA was combined, the survival of patients with negative expression for both or positive for one was four times longer than those with a positive status for both (27.9 months vs. 7.0 months, respectively, p = 0.003, log rank test). Among the several survival predictors by the univariate analysis (T-stage; p = 0.006, tumour differentiation; p = 0.003, metastatic status; p = 0.000), by Cox regression analysis only the combined PKM2 / LDHA expression status and tumour differentiation status were independent survival predictors (p = 0.003, Hazard ratio (HR) = 4.96 and p = 0.015, HR = 3.31, respectively) (Table in S1 Table).

Additionally, 27 of the 72 patients (UCLH cohort) underwent surgical resection for PDAC (n = 16) or ampullary adenocarcinoma (n = 11). Those who underwent surgical resection had a significantly longer survival than those who did not undergo surgery (26.5 vs 7.0 months, P<0.0001; log rank test). As expected, patients with ampullary adenocarcinoma had a better prognosis after surgery than those with PDAC, with 5 of 11 (45.5%) ampullary carcinoma patients alive at last contact, compared with only 1 out of 16 (6.3%) patients with PDAC (P<0.0001; log rank test).

## Discussion

There is mounting evidence that cancer cells have elevated glucose uptake with a concomitant increase in lactate production through sequential catalytic enzyme mediated processes [44,45]. PKM2 and LDHA are two crucial glycolytic enzymes that facilitate these processes to confer cancer cells with a growth advantage over normal cells. To date, serum PKM2 has been identified as a diagnostic and prognostic marker with comparable sensitivity and specificity to serum CA19-9 marker in pancreatic cancer [46–48]. However, there are limited data on the expression pattern and prognostic impact of PKM2 and LDHA in pancreatic cancer [32,49,50]. To the best of our knowledge, our study is the first to evaluate the prognostic impact of combined PKM2 and LDHA expression in the initiation and progression of pancreatic cancer. A recent study showed overexpression and phosphorylation of both of these enzymes in thyroid cancer compared with benign goitre [51]. Our results concur with their findings showing significant overexpression of PKM2 and LDHA in pancreatic cancers compared with normal pancreatic tissue.

The step-wise initiation and development of pancreatic cancer often begins with pancreatitis, ductal metaplasia, cyst formation or PanIN lesions, leading to pancreatic cancer. Interestingly, our results demonstrate overexpression of LDHA at a very early stage along the carcinogenetic pathway from pancreatitis through cyst/PanIN to cancer with the highest expression in the most aggressive tumours. In contrast, PKM2 expression increased progressively along the transition to pancreatic cancer and was lowest in cysts, intermediate in PanIN lesions and highest in cancers. Although the exact mechanism of this differential expression pattern remains to be elucidated, it is possible that the pre-neoplastic lesions acquire the glycolytic phenotype through LDHA overexpression and then LDHA itself or other oncogenes induce PKM2 overexpression at later stages when tumour cell proliferation rates are higher. In fact, it has been recently shown that the epidermal growth factor receptor (EGFR) induces β-catenin transactivation and c-myc expression, upregulating LDHA, which in turn induces upregulation of PKM2 expression by alternating splicing of the gene from M1 to M2 type [52]. These findings might partially explain the consistent overexpression of LDHA throughout the tumorigenic process and the progressive overexpression of PKM2 along the carcinogenetic pathway. The expression and enzymatic activity of LDHA and PKM2 can also be modulated by tyrosine phosphorylation at various residues (Y10 and Y105, respectively) by the oncogenic tyrosine kinase fibroblast growth factor receptor-1 [53]. The differential expression pattern suggests that PKM2 (or a combination of PKM2 and LDHA) would be a better choice for discriminating cancer from pre-neoplastic lesions compared with LDHA alone, except in pancreatitis where both markers are highly expressed.

Treating pancreatic cancer is highly challenging due to late diagnosis, and lack of appropriate prognostic markers and effective therapies. Our results demonstrate that both PKM2 or LDHA are significant prognostic markers in pancreatic cancer and the combination provides improved stratification of outcome. These results are in line with previous publications showing a significant prognostic effect of LDHA or PKM2 in other tumour types, including squamous cell carcinoma, cholangiocarcinoma and gastric cancer [43,54,55]. The exact mechanisms associated with the overexpression of PKM2 and LDHA that lead to poor prognosis remain unclear. Very recently, Rajeshkumar et al (2015) found that the LDHA small molecule inhibitor FX11 can impede tumour growth, reduce tumour cell proliferation and induce apoptosis in a patient-derived mouse xenograft model of pancreatic cancer with mutant TP53, while tumours harbouring wild-type TP53 were completely resistant to FX11 [56].

Pancreatic cancer is one of the more aggressive tumour types, with a very poor overall survival. The results of our study clearly show up-regulation of both PKM2 and LDHA in pancreatic cancer and implicate the high expression of these two glycolytic enzymes in the development and progression of pancreatic cancer through enhanced proliferation, migration, invasion and angiogenesis. Feng et al (2014) reported that the knockdown of PKM2 in pancreatic cancer cells reduces cell proliferation, migration and invasion of pancreatic cancer cells in vitro and induces apoptosis by increasing the expression level of BAX protein and reducing the expression level of Bcl-2 protein [57]. In a related study, Azoitei et al (2013) demonstrated that the knockdown of PKM2 by siRNA attenuates pancreatic cancer cell proliferation and migration in vitro and impedes tumour growth, angiogenesis and Ki67 expression in the pancreatic cancer xenograft model [58].

In this study, we noticed a significant direct correlation between staining scores and the tumour cell proliferation index, and by using the double labeling technique, we were able to identify Ki-67 positive proliferating cells topographically co-localising in PKM2 positive areas. It has been well recognized that these glycolytic enzymes translocate to the nucleus and interact with several other oncogenic transcription factors and transcribe several cell proliferating signaling pathways including Stat3, β-catenin, HIF-1, Oct-4, cyclin D1 [42]. Moreover, PKM2 has been implicated in the phosphorylation of the chromosomal spindle checkpoint protein Bub3-Bub1-Blinkin complex, which ensures fidelity of chromosomal segregation during cell proliferation [59]. Although we could not find any significant correlation with tumour stage and metastasis status, strong expression of PKM2 was observed in metastatic tumours invading muscle and blood vessels, indicating the aggressive phenotype of pancreatic tumours expressing the glycolytic enzymes. We and others have previously shown in cholangiocarcinoma and lung cancer, respectively, that tumour-associated angiogenesis is induced by PKM2 and LDHA, which could be another contributing factor to poor prognosis [43,60].

Host immune evasion is a hallmark of the aggressive tumour phenotype. Although a limited number of studies have provided evidence of mechanism between host immune suppression and the tumour glycolytic phenotype, to date, there is no concrete evidence showing a correlation between the expression of PKM2, LDHA and CD8+ effector cell infiltration. Interestingly, we noticed a significant inverse correlation between the PKM2 and LDHA expression and CD8+ cell infiltration, with an accumulation of CD8+ cells in tumours that did not express PKM2. Recently, Crane et al (2014) reported that LDHA secreted by glioblastoma cells downregulated the Natural Killer group 2, member D receptor on natural killer cells and thus subverted host immune surveillance [61]. Similarly, Liu et al (2015) reported that PKM2 expression was related to increased infiltration of primary and metastatic tumours by myeloid derived suppressor cells, responsible for the suppression of NK cells and induction of host immune suppression by regulatory T cells [62]. We postulate that the compromised immune surveillance induced by enhanced expression of PKM2 and LDHA, and reduced CD8+ effector T-cells might be another contributing factor associated with poor prognosis of the glycolytic phenotype of pancreatic cancer.

Limitations of our study include limited sample size and lack of some clinicopathological information. An optimal scoring system for PKM2 and LDHA expression has not yet been established, with various scoring systems having been reported [43,63–66]. In the current study, PKM2 and LDHA expression scores were classified as negative or positive, which was based on the intensity and extent of PKM2 and LDHA expression across tumour areas. Moreover, other treatments such as chemotherapy might have affected the levels of PKM2 and LDHA expression and overall survival of patients.

In conclusion, we have shown a differential expression pattern for PKM2 and LDHA from cysts through PanIN lesions to pancreatic cancer, with upregulation of LDHA throughout the carcinogenetic process and a progressive upregulation of PKM2 expression along the carcinogenetic pathway. Moreover, the combined expression of these glycolytic enzymes is a strong independent marker of poor prognosis, attributable to increased cell proliferation, larger tumour size and host immune evasion. Further studies are underway to evaluate these markers as possible targets for therapy in pre-clinical models of pancreatic cancer.

## Supporting Information

S1 Table Multivariable analysis of prognostic factors. (DOC)

## References

[1] American Cancer Society. Cancer Fact and Figures 2014. Accessed 16 Dec 2014. Available: http://www.cancer.org/research/cancerfactsfigures/cancerfactsfigures/cancer-facts-figures-2014. 2014.

[2] Cancer Research UK. Pancreatic Cancer Statistics. Accessed 20 Feb 2015. Available: http://www.cancerresearchuk.org/cancer-info/cancerstats/types/pancreas/. 2014.

[3] Conroy T, Desseigne F, Ychou M, Bouché O, Guimbaud R, Bécouarn Y, et al FOLFIRINOX versus gemcitabine for metastatic pancreatic cancer. N Engl J Med. 2011;364(19):1817–25. 10.1056/NEJMoa1011923 21561347

[4] Gourgou-Bourgade S, Bascoul-Mollevi C, Desseigne F, Ychou M, Bouché O, Guimbaud R, et al Impact of FOLFIRINOX compared with gemcitabine on quality of life in patients with metastatic pancreatic cancer: Results from the PRODIGE 4/ACCORD 11 randomized trial. J Clin Oncol. 2013;31(1):23–9. 10.1200/JCO.2012.44.4869 23213101

[5] Wong N, Ojo D, Yan J, Tang D. PKM2 contributes to cancer metabolism. Cancer Letters. 2015;656 (2): 184–191. 10.1016/j.canlet.2014.01.031 24508027

[6] Iqbal MA, Gupta V, Gopinath P, Mazurek S, Bamezai RNK. Pyruvate kinase M2 and cancer: an updated assessment. FEBS Lett. Federation of European Biochemical Societies; 2014; 588(16):2685–92. 10.1016/j.febslet.2014.04.011 24747424

[7] Wang H-J, Hsieh Y-J, Cheng W-C, Lin C-P, Lin Y, Yang S-F, et al JMJD5 regulates PKM2 nuclear translocation and reprograms HIF-1α-mediated glucose metabolism. Proc Natl Acad Sci U S A. 2014; 111(1):279–84. 10.1073/pnas.1311249111 24344305 PMC3890888

[8] Warner S, Carpenter K, Bearss D. Activators of PKM2 in cancer metabolism. Future Med Chem. 2014;06:1167–78. 10.4155/fmc.14.70 25078136

[9] Gao Xueliang, Wang Haizhen, Jenny J. Yang, Liu Xiaowei and Z-RL. Pyruvate Kinase M2 Regulates Gene Transcription by Acting as A Protein Kinas. 2012;45(5):598–609. 10.1016/j.molcel.2012.01.001 PMC3299833 22306293

[10] Mazurek S. Pyruvate kinase type M2: a key regulator of the metabolic budget system in tumor cells. Int J Biochem Cell Biol. 2011;43(7):969–80. 10.1016/j.biocel.2010.02.005 20156581

[11] Kumar Y, Mazurek S, Yang S, Failing K, Winslet M, Fuller B, et al In vivo factors influencing tumour M2-pyruvate kinase level in human pancreatic cancer cell lines. Tumour Biol. 2010;31(2):69–77. 10.1007/s13277-009-0010-3 20358419

[12] Vander Heiden MG, Christofk HR, Schuman E, Subtelny AO, Sharfi H, Harlow EE, et al Identification of small molecule inhibitors of pyruvate kinase M2. Biochem Pharmacol. Elsevier Inc.; 2010; 79(8):1118–24. 10.1016/j.bcp.2009.12.003 20005212 PMC2823991

[13] Christofk HR, Vander Heiden MG, Harris MH, Ramanathan A, Gerszten RE, Wei R, et al The M2 splice isoform of pyruvate kinase is important for cancer metabolism and tumour growth. Nature. 2008;452(7184):230–3. 10.1038/nature06734 18337823

[14] Jurica MS, Mesecar a, Heath PJ, Shi W, Nowak T, Stoddard BL. The allosteric regulation of pyruvate kinase by fructose-1,6-bisphosphate. Structure. 1998;6(2):195–210. 9519410 10.1016/s0969-2126(98)00021-5

[15] Kumar Y, Tapuria N, Kirmani N, Davidson BR. Tumour M2-pyruvate kinase: a gastrointestinal cancer marker. Eur J Gastroenterol Hepatol. 2007;19(3):265–76. 17301655 10.1097/MEG.0b013e3280102f78

[16] Li YG, Zhang N. Clinical significance of serum tumour M2-PK and CA19-9 detection in the diagnosis of cholangiocarcinoma. Dig Liver Dis. 2009;41(8):605–8. 10.1016/j.dld.2008.11.010 19168405

[17] Mazurek S, Boschek CB, Hugo F, Eigenbrodt E. Pyruvate kinase type M2 and its role in tumor growth and spreading. Semin Cancer Biol. 2005;15(4):300–8. 15908230 10.1016/j.semcancer.2005.04.009

[18] Hardt P. D., and Ewald N. Tumor M2 pyruvate kinase: a tumor marker and its clinical application in gastrointestinal malignancy. Expert Rev Mol Diagn. 2008;8:579–85. 10.1586/14737159.8.5.579 18785806

[19] Spoden G a, Rostek U, Lechner S, Mitterberger M, Mazurek S, Zwerschke W. Pyruvate kinase isoenzyme M2 is a glycolytic sensor differentially regulating cell proliferation, cell size and apoptotic cell death dependent on glucose supply. Exp Cell Res. Elsevier Inc.; 2009;315(16):2765–74. 10.1016/j.yexcr.2009.06.024 19563799

[20] Miao P, Sheng S, Sun X, Liu J, Huang G. Lactate dehydrogenase a in cancer: A promising target for diagnosis and therapy. IUBMB Life. 2013 p. 904–10. 10.1002/iub.1216 24265197

[21] Doherty JR, Cleveland JL. Targeting lactate metabolism for cancer therapeutics. J Clin Invest. 2013;123(9):3685–92. 10.1172/JCI69741 23999443 PMC3754272

[22] Fiume L, Vettraino M, Stefano G Di. Inhibition of lactate dehydrogenase activity as an approach to cancer therapy. 2014;429–45. 10.4155/fmc.13.206 24635523

[23] Le A, Cooper CR, Gouw AM, Dinavahi R, Maitra A, Deck LM, et al Inhibition of lactate dehydrogenase A induces oxidative stress and inhibits tumor progression. Proc Natl Acad Sci U S A. 2010;107(5):2037–42. 10.1073/pnas.0914433107 20133848 PMC2836706

[24] Billiard J, Dennison JB, Briand J, Annan RS, Chai D, Colón M, et al Quinoline 3-sulfonamides inhibit lactate dehydrogenase A and reverse aerobic glycolysis in cancer cells. Cancer Metab. 2013;1(1):19 10.1186/2049-3002-1-19 24280423 PMC4178217

[25] Yu Y, Deck JA, Hunsaker LA, Deck LM, Royer RE, Goldberg E, et al Selective active site inhibitors of human lactate dehydrogenases A4, B4, and C4. Biochem Pharmacol. 2001;62(1):81–9. 11377399 10.1016/s0006-2952(01)00636-0

[26] Xue J-J, Chen Q-Y, Kong M-Y, Zhu C-Y, Gen Z-R, Wang Z-L. Synthesis, cytotoxicity for mimics of catalase: Inhibitors of lactate dehydrogenase and hypoxia inducible factor. Eur J Med Chem. Elsevier; 2014;80:1–7. 10.1016/j.ejmech.2014.04.035 24763359

[27] Koukourakis MI, Giatromanolaki A, Sivridis E, Bougioukas G, Didilis V, Gatter KC, et al Lactate dehydrogenase-5 (LDH-5) overexpression in non-small-cell lung cancer tissues is linked to tumour hypoxia, angiogenic factor production and poor prognosis. Br J Cancer. Nature Publishing Group; 2003; 89(5):877–85. 12942121 10.1038/sj.bjc.6601205 PMC2394471

[28] Le A, Rajeshkumar N V, Maitra A, Dang C V. Conceptual framework for cutting the pancreatic cancer fuel supply. Clin Cancer Res. 2012;18(16):4285–90. 10.1158/1078-0432.CCR-12-0041 22896695 PMC3545437

[29] Zhao D, Xiong Y, Lei Q-Y, Guan K-L. LDH-A Acetylation: implication in Pancreatic Cancer Initiation and Diagnosis. Oncotarget. 2013; 4(6):802–803. 23868819 10.18632/oncotarget.1007 PMC3757233

[30] Yu Y, Liao M, Liu R, Chen J, Feng H, Fu Z. Overexpression of lactate dehydrogenase-A in human intrahepatic cholangiocarcinoma: its implication for treatment. World J Surg Oncol. 2014;12:78 10.1186/1477-7819-12-78 24679073 PMC4230420

[31] Zhou C, Li X, Sun H, Zhang B, Han Y, Jiang Y, et al Pyruvate kinase type M2 is upregulated in colorectal cancer and promotes proliferation and migration of colon cancer cells. IUBMB Life. Wiley Online Library; 2012;64(9):775–82. 10.1002/iub.1066 22807066

[32] Rong Y, Wu W, Ni X, Kuang T, Jin D, Wang D, et al Lactate dehydrogenase A is overexpressed in pancreatic cancer and promotes the growth of pancreatic cancer cells. Tumour Biol. 2013;34(3):1523–30. 10.1007/s13277-013-0679-1 23404405

[33] Anastasiou D, Yu Y, Israelsen WJ, Jiang J, Boxer MB, Hong BS, et al Pyruvate kinase M2 activators promote tetramer formation and suppress tumorigenesis. Nat Chem Biol. 2012 10;8(10):839–47. 22922757 10.1038/nchembio.1060 PMC3711671

[34] Fukunaga A, Miyamoto M, Cho Y, Murakami S, Kawarada Y, Oshikiri T, et al CD8+ tumor-infiltrating lymphocytes together with CD4+ tumor-infiltrating lymphocytes and dendritic cells improve the prognosis of patients with pancreatic adenocarcinoma. Pancreas. LWW; 2004;28(1):e26–31. 14707745 10.1097/00006676-200401000-00023

[35] Kawai O, Ishii G, Kubota K, Murata Y, Naito Y, Mizuno T, et al Predominant infiltration of macrophages and CD8+ T cells in cancer nests is a significant predictor of survival in stage IV nonsmall cell lung cancer. Cancer. Wiley Online Library; 2008;113(6):1387–95. 10.1002/cncr.23712 18671239

[36] Sato E, Olson SH, Ahn J, Bundy B, Nishikawa H, Qian F, et al Intraepithelial CD8+ tumor-infiltrating lymphocytes and a high CD8+/regulatory T cell ratio are associated with favorable prognosis in ovarian cancer. Proc Natl Acad Sci U S A. National Acad Sciences; 2005;102(51):18538–43. 16344461 10.1073/pnas.0509182102 PMC1311741

[37] Pagès F, Kirilovsky A, Mlecnik B, Asslaber M, Tosolini M, Bindea G, et al In situ cytotoxic and memory T cells predict outcome in patients with early-stage colorectal cancer. J Clin Oncol. American Society of Clinical Oncology; 2009;27(35):5944–51. 10.1200/JCO.2008.19.6147 19858404

[38] Nakano O, Sato M, Naito Y, Suzuki K, Orikasa S, Aizawa M, et al Proliferative activity of intratumoral CD8+ T-lymphocytes as a prognostic factor in human renal cell carcinoma clinicopathologic demonstration of antitumor immunity. Cancer Res. AACR; 2001;61(13):5132–6. 11431351

[39] Ashida A, Boku N, Aoyagi K, Sato H, Tsubosa Y, Minashi K, et al Expression profiling of esophageal squamous cell carcinoma patients treated with definitive chemoradiotherapy: clinical implications. Int J Oncol. Spandidos Publications; 2006;28(6):1345–52. 16685435

[40] Ikeguchi M, Oi K, Hirooka Y, Kaibara N. CD8+ lymphocyte infiltration and apoptosis in hepatocellular carcinoma. Eur J Surg Oncol. 2004;30(1):53–7. 14736523 10.1016/j.ejso.2003.10.001

[41] Mahmoud SM a, Paish EC, Powe DG, Macmillan RD, Grainge MJ, Lee AHS, et al Tumor-infiltrating CD8+ lymphocytes predict clinical outcome in breast cancer. J Clin Oncol. 2011;29(15):1949–55. 10.1200/JCO.2010.30.5037 21483002

[42] Li Z, Yang P, Li Z. The multifaceted regulation and functions of PKM2 in tumor progression. Biochim Biophys Acta. Elsevier B.V.; 2014;1864(2): 285–296. 10.1016/j.bbcan.2014.07.008 25064846

[43] Dhar DK, Olde Damink SWM, Brindley JH, Godfrey A, Chapman MH, Sandanayake NS, et al Pyruvate kinase M2 is a novel diagnostic marker and predicts tumour progression in human biliary tract cancer. Cancer. 2013 2;119(3):575–85. 10.1002/cncr.27611 22864959 PMC3492546

[44] Wong N, De Melo J, Tang D. PKM2, a central point of regulation in cancer metabolism. Int J Cell Biol. 2013;(2013):1–11. 10.1155/2013/242513 PMC3586519 23476652

[45] Singh S, Tan M, Rameshwar P. Cancer Metabolism: Targeting metabolic pathways in cancer therapy. Cancer Lett. 2015;356:147–8. 10.1016/j.canlet.2014.06.002 24956174

[46] Kumar Y, Gurusamy K, Pamecha V, Davidson BR. Tumor M2-pyruvate kinase as tumor marker in exocrine pancreatic cancer a meta-analysis. Pancreas. 2007;35(2):114–9. 17632316 10.1097/mpa.0b013e3180537237

[47] Goonetilleke KS, Mason JM, Siriwardana P, King NK, France MW, Siriwardena AK. Diagnostic and prognostic value of plasma tumor M2 pyruvate kinase in periampullary cancer: evidence for a novel biological marker of adverse prognosis. Pancreas. 2007;34(3):318–24. 17414054 10.1097/MPA.0b013e31802ee9c7

[48] Joergensen MT, Heegaard NHH, Schaffalitzky de Muckadell OB. Comparison of plasma Tu-M2-PK and CA19-9 in pancreatic cancer. Pancreas. 2010;39(2):243–7. 10.1097/MPA.0b013e3181bae8ab 19820423

[49] Shi M, Cui J, Du J, Wei D, Jia Z, Zhang J, et al A novel KLF4/LDHA signaling pathway regulates aerobic glycolysis in and progression of pancreatic cancer. Clin Cancer Res. AACR; 2014;20(16):4370–80. 10.1158/1078-0432.CCR-14-0186 24947925 PMC4134726

[50] He T-L, Zhang Y-J, Jiang H, Li X, Zhu H, Zheng K-L. The c-Myc–LDHA axis positively regulates aerobic glycolysis and promotes tumor progression in pancreatic cancer. Med Oncol. Springer; 2015;32(7):1–8. 10.1007/s12032-015-0633-8 PMC4452209 26021472

[51] Kachel P, Trojanowicz B, Sekulla C, Prenzel H, Dralle H, Hoang-Vu C. Phosphorylation of pyruvate kinase M2 and lactate dehydrogenase A by fibroblast growth factor receptor 1 in benign and malignant thyroid tissue. BMC Cancer. BioMed Central Ltd; 2015;15(1):140. 25880801 10.1186/s12885-015-1135-y PMC4393606

[52] Yang W, Zheng Y, Xia Y, Ji H, Chen X, Guo F, et al ERK1/2-dependent phosphorylation and nuclear translocation of PKM2 promotes the Warburg effect. Nat Cell Biol. Nature Publishing Group; 2012;14(12):1295–304. 10.1038/ncb2629 23178880 PMC3511602

[53] Hitosugi T, Kang S, Vander Heiden MG, Chung T-W, Elf S, Lythgoe K, et al Tyrosine phosphorylation inhibits PKM2 to promote the Warburg effect and tumor growth. Sci Signal. NIH Public Access; 2009;2(97):ra73 10.1126/scisignal.2000431 19920251 PMC2812789

[54] Sun X, Sun Z, Zhu Z, Li C, Zhang J, Xu H, et al Expression of SIP1 is strongly correlated with LDHA and shows a significantly poor outcome in gastric cancer. Tumor Biol. Springer; 2015;1–10. 10.1007/s13277-015-3470-7 25913622

[55] Wang Y, Zhang X, Zhang Y, Zhu Y, Yuan C, Qi B, et al Overexpression of pyruvate kinase M2 associates with aggressive clinicopathological features and unfavorable prognosis in oral squamous cell carcinoma. Cancer Biol Ther. Taylor & Francis; 2015;(just-accepted):0. 10.1080/15384047.2015.1030551 PMC4622565 25970228

[56] Rajeshkumar N V., Dutta P, Yabuuchi S, de Wilde RF, Matrinez G V., Le a., et al Therapeutic targeting of the Warburg effect in pancreatic cancer relies on an absence of p53 function. Cancer Research. 2015 p.713–745. 26113084 10.1158/0008-5472.CAN-15-0108 PMC4537812

[57] Feng J, Ma T, Ge Z, Lin J, Ding W, Chen H, et al PKM2 gene regulates the behavior of pancreatic cancer cells via mitogen-activated protein kinase pathways. Mol Med Rep. Spandidos Publications; 2015;11(3):2111–7. 10.3892/mmr.2014.2990 25411978

[58] Azoitei N, Rehbein G, Genze F, Brobovich S, Armacki M, Fiedler K, et al Role of Pyruvate kinase M2 (PKM2) in tumor growth, cancer cell migration and tumor angiogenesis. Z Gastroenterol. 51(08):K55.

[59] Jiang Y, Li X, Yang W, Hawke DH, Zheng Y, Xia Y, et al PKM2 Regulates Chromosome Segregation and Mitosis Progression of Tumor Cells. Mol Cell. 2014;53(1):75–87. 10.1016/j.molcel.2013.11.001 24316223 PMC3955203

[60] Parra-Bonilla G, Alvarez DF, Alexeyev M, Vasauskas A, Stevens T. Lactate Dehydrogenase A Expression Is Necessary to Sustain Rapid Angiogenesis of Pulmonary Microvascular Endothelium. PLoS One. 2013;8(9). 10.1371/journal.pone.0075984 PMC3784391 24086675

[61] Crane CA, Austgen K, Haberthur K, Hofmann C, Moyes KW, Avanesyan L, et al Immune evasion mediated by tumor-derived lactate dehydrogenase induction of NKG2D ligands on myeloid cells in glioblastoma patients. Proc Natl Acad Sci. 2014; 111(35):12823–12828. 10.1073/pnas.1413933111 25136121 PMC4156766

[62] Liu W-R, Tian M-X, Yang L-X, Lin Y-L, Jin L, Ding Z-B, et al PKM2 promotes metastasis by recruiting myeloid-derived suppressor cells and indicates poor prognosis for hepatocellular carcinoma. Oncotarget. Impact Journals, LLC; 2015;6(2):846 25514599 10.18632/oncotarget.2749 PMC4359260

[63] Lockney NA, Zhang M, Lu Y, Sopha SC, Washington MK, Merchant N, et al Pyruvate Kinase Muscle Isoenzyme 2 (PKM2) Expression Is Associated with Overall Survival in Pancreatic Ductal Adenocarcinoma. J Gastrointest Cancer. 2015; 46 (4): 390–398. 10.1007/s12029-015-9764-6 26385349 PMC7081381

[64] Lim JY, Yoon SO, Seol SY, Hong SW, Kim JW, Choi SH, et al Overexpression of the M2 isoform of pyruvate kinase is an adverse prognostic factor for signet ring cell gastric cancer. World J Gastroenterol. 2012;18:4037–43. 10.3748/wjg.v18.i30.4037 22912555 PMC3420001

[65] Zhang X, He C, He C, Chen B, Liu Y, Kong M, et al Nuclear PKM2 expression predicts poor prognosis in patients with esophageal squamous cell carcinoma. Pathol Res Pract. 2013;209:510–5. 10.1016/j.prp.2013.06.005 23880164

[66] Li J, Yang Z, Zou Q, Yuan Y, Liang L, Zeng G, et al PKM2 and ACVR 1C are prognostic markers for poor prognosis of gallbladder cancer. Clin Transl Oncol. 2014;16:200–7. 10.1007/s12094-013-1063-8 23793810

