# Supplementary material for: Pyruvate Kinase M2 and Lactate Dehydrogenase A Are Overexpressed in Pancreatic Cancer and Correlate with Poor Outcome
Source: PLoS One. 2016 Mar 18;11(3):e0151635. doi: 10.1371/journal.pone.0151635 (PMC4798246; doi:10.1371/journal.pone.0151635)
Supplement: S1 Table — (DOC) [file pone.0151635.s001.doc]

S1 Table. Multivariable analysis of prognostic factors

|  | **Variables in equation** | **p-value** | **Hazard ratio** |
| --- | --- | --- | --- |
| Step 1 | Tumour differentiation | 0.096 | 3.372 |
|  | T-stage | 0.877 | 0.937 |
|  | Metastatic status | 0.223 | 0.333 |
|  | PKM2 and LDHA combined expression | 0.007 | 4.538 |
| Step 2 | Tumour differentiation | 0.024 | 3.109 |
|  | Metastatic status | 0.203 | 0.324 |
|  | PKM2 and LDHA combined expression | 0.005 | 4.642 |
| Step 3 | Tumour differentiation | 0.015 | 3.314 |
|  | PKM2 and LDHA combined expression | 0.003 | 4.959 |
